# Cost-effectiveness of solifenacin compared with oral antimuscarinic agents for the treatment of patients with overactive bladder in the UK

**DOI:** 10.1080/20016689.2018.1438721

**Published:** 2018-03-20

**Authors:** Zalmai Hakimi, Con Kelleher, Samuel Aballéa, Khaled Maman, Jameel Nazir, Colette Mankowski, Isaac Odeyemi

**Affiliations:** a Medical Affairs Department, Astellas Pharma Europe B.V, Leiden, The Netherlands; b Department of Obstetrics and Gynecology, Guy’s and St. Thomas’ NHS Foundation Trust, London, UK; c Health Economics and Outcomes Research Department, Creativ-Ceutical SARL, Paris, France; d Health Economics and Outcomes Research Department, Creativ-Ceutical Ltd, London, UK; e Medical Affairs Department, Astellas Pharma Europe Ltd, Chertsey, UK

**Keywords:** Cost utility, incremental cost-effectiveness ratio, Markov, quality-adjusted life year, mirabegron, muscarinic agents

## Abstract

**Objective:** To evaluate the cost-effectiveness of solifenacin 5 mg/day versus other oral antimuscarinic agents used for overactive bladder (OAB) from a UK National Health Service (NHS) perspective. **Study design:** In a Markov model, hypothetical patients received solifenacin 5 mg/day or a comparator antimuscarinic, after which they could switch to an alternative antimuscarinic. The model estimated incremental cost-effectiveness ratios (ICER), expressed as cost per quality-adjusted life year (QALY) over a 5-year period. **Results:** Solifenacin 5 mg/day was the dominant treatment strategy (i.e., less costly and more effective) versus tolterodine extended-release (ER) 4 mg/day, fesoterodine 4 and 8 mg/day, oxybutynin ER 10 mg/day and solifenacin 10 mg/day, and was cost-effective (i.e., ICERs below the £30,000 per QALY threshold generally applied in the NHS) versus oxybutynin immediate release (IR) 10 mg/day, tolterodine IR 4 mg/day and trospium chloride 60 mg/day. The probability of solifenacin 5 mg/day being dominant/cost-effective at a willingness-to-pay threshold of £30,000 per QALY was 57–98%. **Conclusions:** Solifenacin 5 mg/day appears to be a cost-effective strategy for the treatment of OAB over a 5-year timeframe compared with other oral antimuscarinic agents in the UK. These findings are important for decision-makers considering the economic implications of selecting treatments for OAB.

## Introduction

Overactive bladder (OAB) is characterized by urinary urgency, which is usually accompanied by frequency and nocturia, with or without urinary incontinence [1]. Symptoms experienced by patients with OAB can profoundly and negatively affect many aspects of health-related quality of life (HRQoL), including psychological, social, occupational, and sexual function [2]. Individuals with OAB report significantly lower HRQoL scores compared with matched controls, indicating impaired overall health [2].

OAB is a common condition and its prevalence increases with age [3]. The overall global prevalence of OAB was 10.7% (455 million cases) in 2008, and a 20.1% increase is predicted by 2018 (546 million cases) [4]. The associated costs of OAB are substantial. An economic model estimated that the annual cost of OAB to the UK healthcare system was approximately €1 billion (2005 values), with an additional €579 million required for nursing home stays and €233 million for lost productivity due to work absenteeism [5].

In the UK, the National Institute for Health and Care Excellence (NICE) recommends conservative management (e.g., bladder training and lifestyle advice) as initial treatment for OAB or storage lower urinary tract symptoms, followed by pharmacotherapy with antimuscarinic agents [6,7] or the β_3_-adrenoceptor agonist, mirabegron [7]. Several antimuscarinics are approved for treating OAB, including older generation (e.g., oxybutynin and trospium chloride) and newer generation (e.g., solifenacin, tolterodine, and fesoterodine) agents.

Solifenacin is a competitive muscarinic M_3_-receptor antagonist available as an oral once-daily tablet at two dosage strengths [8]. The efficacy of solifenacin 5 and 10 mg (fixed doses) and 5 mg/10 mg (flexible dosing schedule) has been comprehensively studied in a series of large randomized controlled trials (RCTs) in patients with OAB [9–12]. These show that solifenacin is significantly more effective than placebo [9,10], and provides greater efficacy than tolterodine for most outcomes [9,11,12]. The most commonly reported adverse events (AEs) with solifenacin are dry mouth and constipation [9,10].

OAB is a chronic condition and long-term use of pharmacotherapy may be required to control symptoms. Although many cost-effectiveness analyses of antimuscarinic agents in OAB have been performed, most compare only two agents over a short time-frame based on available data from RCTs and therefore provide only limited economic data to guide clinical decision-makers. By applying data from a recent network meta-analysis (NMA) [13] to a Markov model for OAB [14,15], we constructed a single analysis to evaluate the long-term (5-year) cost-effectiveness of solifenacin 5 mg/day compared with several other oral antimuscarinics routinely prescribed for adults with OAB from a UK National Health Service (NHS) perspective.

## Materials and methods

### Model overview

Markov modelling is a decision-tree method frequently used to evaluate the cost-effectiveness of medicines, where the disease or treatment process is represented by a series of health states. Markov models assume that a patient is always in one of a finite number of discrete health states (e.g., severity of symptoms) and the effect of treatment is represented as transitions between states [16]. The models are set to a clinically relevant time horizon, divided into equal segments (i.e., cycles); during each cycle, patients have a defined probability of remaining in the same state or transitioning between states. Each health state is associated with a specific cost and utility value (i.e., relative preference scores between different health states on a scale of 0 [worst imaginable health] to 1 [full/perfect health]). Cumulative costs and quality-adjusted life years (QALYs; i.e., health-related benefit gained) for each treatment at the designated time horizon are calculated.

### Model description

A prior Markov model was developed to simulate the therapeutic management and disease course in a hypothetical cohort of adults with OAB [14,15]. The model was adapted to predict the costs and QALYs gained from treatment with solifenacin 5 mg/day versus other oral antimuscarinics. The base-case scenario included a 1-month cycle and a 5-year time horizon. The model was programmed in Microsoft® Excel 2010. Costs and outcomes were discounted at a rate of 3.5% per year [17], which converts future expected values to current values.

The model and assumptions applied were described previously in detail [14,15]. The model simulates changes in key OAB symptoms (i.e., number of micturitions and incontinence episodes per day), transitions between treatments (i.e., initial treatment, next-line treatment, off-treatment, and botulinum toxin type A [BTX-A]); and occurrence of AEs (i.e., dry mouth and constipation). Different antimuscarinic agents are characterized by their effects on symptoms, likelihood of AEs, and acquisition costs.

At model entry, patients were assigned to either oral solifenacin 5 mg/day or a comparator antimuscarinic agent (Figure S1). For the primary analysis, the comparator was tolterodine extended-release (ER) 4 mg/day; for the secondary analyses, the comparators were fesoterodine 4 or 8 mg/day, oxybutynin ER or immediate-release (IR) 10 mg/day, solifenacin 10 mg/day, tolterodine IR 4 mg/day, or trospium chloride 60 mg/day. BTX-A (injection into the detrusor muscle) was an option for patients who failed treatment with ≥2 antimuscarinics.

### Model inputs

#### Key data sources

Data for solifenacin 5 mg/day were derived from 905-CL-015, an international, multicenter, double-blind, controlled trial that randomized patients (n = 1081) to 12 weeks’ treatment with solifenacin 5 or 10 mg/day, tolterodine 4 mg/day, or placebo [10]. Solifenacin (5 and 10 mg/day) was reported as significantly more effective than placebo in decreasing the frequency of urgency, urgency urinary incontinence, all incontinence and micturition episodes over 24 hours. There was no formal statistical testing of solifenacin versus tolterodine.

In the absence of direct head-to-head trials, NMAs are an extension of traditional meta-analyses and allow multiple pairwise comparisons across interventions [18]. We conducted a Bayesian NMA to estimate the relative efficacy and tolerability of solifenacin 5 mg/day versus other oral antimuscarinics in patients with OAB [13]. The NMA included 53 RCTs, identified from a systematic literature search performed in 2015 [13]; the NMA data used in our model are presented in Table S1.

#### Symptom severity

Micturition frequency and incontinence had five severity levels in the model. The initial distribution of patients in each symptom severity stratum was based on pooled baseline data from trial 905-CL-015 (Table S2). From months 0 to 3, monthly transition probabilities between symptom levels for solifenacin 5 mg/day were estimated by applying a multinomial regression model to the 905-CL-015 data (Table S3). Transition probabilities after Month 3 were assumed to be the same as between Months 2 and 3, based on evidence from two long-term studies which suggested that the treatment effect of antimuscarinic agents at 3 or 4 months is sustained up to 24 months [19,20]. For other antimuscarinics, transition probabilities were derived from the NMA results using a calibration approach [21], which aimed to minimize the difference between changes in symptom severity predicted from the model and changes estimated from the NMA. Transition probabilities for next-line therapies were assumed to be the same as for tolterodine. After treatment discontinuation, patient distribution by symptom severity was assumed to be similar to baseline.

#### Adverse events

The probabilities of dry mouth and constipation for solifenacin 5 mg/day were derived from 905-CL-015 [10,22]. For other antimuscarinics, odds ratios from the NMA [13] (Table S1) were used to adjust the probability of each AE (Table 1).

**Table 1.** Model inputs for adverse events, treatment pathways, and utilities.

Parameter	Base-case value	Deterministic sensitivity analysis values	Source
**Adverse events, %^a^**			
Dry mouth			
Solifenacin 5 mg	14.3	10.2–18.4	Astellas [22]
Tolterodine ER 4 mg	14.9	12.8–17.8	Nazir et al. [13]
Fesoterodine 4 mg	15.1	12.2–19.3	Nazir et al. [13]
Fesoterodine 8 mg	28.9	24.8–33.7	Nazir et al. [13]
Oxybutynin ER 10 mg	21.0	16.3–27.2	Nazir et al. [13]
Oxybutynin IR 10 mg	37.1	26.4‒51.0	Nazir et al. [13]
Solifenacin 10 mg	27.5	24.3–30.9	Nazir et al. [13]
Tolterodine IR 4 mg	22.3	18.1–25.7	Nazir et al. [13]
Trospium chloride 60 mg	12.7	7.7‒18.5	Nazir et al. [13]
Constipation			
Solifenacin 5 mg	7.2	4.2–10.2	Chapple et al. [10]
Tolterodine ER 4 mg	4.3	3.0–6.2	Nazir et al. [13]
Fesoterodine 4 mg	3.7	2.4–5.8	Nazir et al. [13]
Fesoterodine 8 mg	6.9	4.8‒10	Nazir et al. [13]
Oxybutynin ER 10 mg	3.7	1.7–7.5	Nazir et al. [13]
Oxybutynin IR 10 mg	3.7	2.1–6.4	Nazir et al. [13]
Solifenacin 10 mg	12.2	9.9–15	Nazir et al. [13]
Tolterodine IR 4 mg	3.7	1.7–7.5	Nazir et al. [13]
Trospium chloride 60 mg	15.1	8.1‒27.7	Nazir et al. [13]
**Treatment pathways**			
Monthly probability of treatment discontinuation, %			
Without adverse events			
Solifenacin 5 mg/day	6.8	3.4–10.2	Calculated from Chapple et al. [24] and Castro-Diaz et al. [23]
Other antimuscarinics	6.8	3.4–10.2	Assumption
With adverse events	90.0	50.0–100.0	Assumption
Monthly probability of treatment switch after discontinuation, %	26.1	6.7–39.1	Odeyemi et al. [25];^b^ assumption
Monthly probability of treatment restart, %	5.6	0–8.42	Assumption^c^
Monthly probability of BTX-A injection, %	0.1	0–0.05	Assumption
Probability of reinjection after 6 months, %	70.0	50.0–100.0	Assumption
**Utilities^d^**			
Micturition			Aballéa et al. [14]
Level 1	0.0632	0.0453–0.0811	
Level 2	0.0422	0.0258–0.0587	
Level 3	0.0204	0.0045–0.0363	
Level 4	0.0104	–0.0316	
Incontinence			Aballéa et al. [14]
Level 1	0.0586	0.0422–0.0749	
Level 2	0.0437	0.0271–0.0602	
Level 3	0.0314	0.0142–0.0486	
Level 4	0.0128	–0.0369	
Decrement for adverse events	–0.0357	0 to – 0.1	Aballéa et al. [14]

BTX-A, botulinum toxin type A; ER, extended release; IR, immediate release.

^a^Probability at 3 months. ^b^Based on switching among patients on tolterodine. ^c^A monthly probability of 5.6% (50% annually) was assumed for restarting treatment among patients who discontinued treatment without immediately switching to another drug. It was assumed that one-third of these patients would go back to their previous treatment, and one-third each would receive next line A and next line B. ^d^Coefficients for symptom severity level relative to symptom level 5, e.g. the utility of patients at micturition severity level 1 is higher than the utility of patients with micturition severity level 5 by 0.0632.

#### Treatment discontinuation, switch and restart

For the base-case analysis, the discontinuation rate was disaggregated according to whether patients withdrew because of AEs (i.e., dry mouth or constipation) or other reasons (i.e., lack of efficacy or other) (Table 1). The probability of discontinuation due to AEs was assumed to be 90% as no published data were available for this parameter. The probability of discontinuation for other reasons (6.8%) was estimated from the literature [23,24]. The probability of treatment switch after discontinuation (26.1%) was taken from a UK study [25], and the probability of restarting treatment among patients who discontinued treatment without immediate treatment switch was assumed in the absence of published data (5.6%).

#### Botulinum toxin type A

The probability of receiving BTX-A and treatment success with BTX-A were assumed in the absence of published data (Table 1). If BTX-A treatment was successful, it was assumed that injections were repeated 6-monthly, whereas patients who failed BTX-A were assumed to receive no further treatment.

#### Health-state utilities

Health-state utility values according to symptom severity were derived from EuroQol five dimensions questionnaire (EQ-5D) index scores, a generic HRQoL instrument, reported in the SCORPIO trial [26], using a UK time trade-off tariff [27]. A linear regression model was used to estimate EQ-5D utilities by symptom severity (Table 1), adjusting for independent variables of patient sex, age and country (Table S4). A utility decrement for AEs was also estimated based on repeated regression analysis of the EQ-5D data (Table 1); this was applied for the duration of a cycle for patients who experienced AEs and stayed on-treatment.

#### Costs and resource utilization

Costs and resources were considered from a UK NHS perspective (Table 2) [28–31]. Direct medical costs and resources considered in the model were: *antimuscarinic drug acquisition costs*: patients were assumed to use 1 tablet/day until discontinuation (drug wastage and partial compliance were not considered); *primary care and specialist consultations*: visits were assumed at baseline and upon initiation of a new medication; *specialist consultations*: 1.5 outpatient urologist visits were assumed upon initiation of new medication [32]; *incontinence pads*: mean number of pads/day was based on incontinence severity level and estimated using data from 905-CL-015 [33]; and *BTX-A injections*: acquisition and administration costs [28–30]. Costs were presented in pounds sterling (2015–2016 values).

**Table 2.** Model inputs for resource use and costs.

Parameter	Base-case value	Deterministic sensitivity analysis values	Source
**Resource use**			
Pad utilization, number per month^a^			Astellas [33]
Level 1	6.97	5.83–8.10	
Level 2	23.48	20.80–26.16	
Level 3	44.47	39.76–49.18	
Level 4	58.13	52.10–64.15	
Level 5	121.30	111.88–130.72	
GP consultations	1 visit at treatment initiation and BTX-A;	0‒2	Assumption
Specialist consultations	1 visit at treatment initiation and BTX-A;	0‒2	Assumption
**Costs**			
Monthly acquisition costs			
Solifenacin 5 mg	£28.00	‒	BNF [28]
Tolterodine ER 4 mg	£28.01	‒	BNF [28]
Fesoterodine 4 mg	£28.01	‒	BNF [28]
Fesoterodine 8 mg	£28.01	‒	BNF [28]
Oxybutynin ER 10 mg	£27.92	‒	BNF [28]
Oxybutynin IR 10 mg	£2.40	‒	BNF [28]
Solifenacin 10 mg	£36.41	‒	BNF [28]
Tolterodine IR 4 mg	£2.88	‒	BNF [28]
Trospium chloride 60 mg	£25.04	‒	BNF [28]
GP consultation (first visit)	£65.00	‒	Curtis & Burns [29]
GP consultation (follow-up)	£27.00	‒	Curtis & Burns [29]
Specialist visit (first visit or follow-up)	£94.00	‒	Department of Health [30]
BTX-A injection/reinjection	£1,151.98^b^	‒	BNF [28]; Curtis & Burns [29]; Department of Health [30]
Incontinence pad	£0.17	‒	AgeUK incontinence [31]

BTX-A, botulinum toxin type A; ER, extended release; GP, general practitioner; IR, immediate release. ^a^According to incontinence symptom severity level. ^b^Botox® Allergan 100 unit vial + intermediate endoscopic bladder procedure + nurse review.

### Model outputs

Cost-effectiveness was estimated by calculating the relative differences in total costs and QALYs for solifenacin 5 mg/day versus another antimuscarinic over the model time horizon to yield an incremental cost-effectiveness ratio (ICER), expressed as cost per QALY gained.

$$ICER\;=\;\frac{Cost\;\;difference\;\;(solifenacin\;\;5\;mg/day\;-\;comparator\;\;antimuscarinic)}{QALY\;\;difference\;(solifenacin\;\;5\;mg/day\;-\;comparator\;\;antimuscarinic)}$$

A willingness-to-pay (WTP) threshold of £30,000 per QALY gained was used to interpret the ICERs in this study. A maximum acceptable ICER threshold of £20,000–£30,000 per QALY gained is generally used to determine the probability that a treatment is cost effective in the UK [17], but reports suggest that some decisions appear to have been based on a threshold higher than £20,000–£30,000 per QALY gained [34].

### Sensitivity and scenario analyses

Model inputs are subject to uncertainty; therefore, the stability of the base-case results were tested using deterministic and probabilistic sensitivity analyses. For the deterministic sensitivity analysis, model parameters were varied independently over a plausible range of values determined either by using the 95% confidence intervals around the point estimate or a sensible range of values where there was no sampling uncertainty (Table 1). Data were depicted in Tornado diagrams. For the probabilistic sensitivity analysis, input variables with uncertainties were varied simultaneously according to predefined statistical distributions (data not shown). One thousand iterations with different sets of inputs drawn randomly from the statistical distributions were depicted in a cost-effectiveness plane, and the probability that solifenacin 5 mg/day was cost effective at different WTP thresholds was shown in a cost-effectiveness acceptability curve. A scenario analysis, in which discontinuation rates for each antimuscarinic agent were derived from a recent UK observational study [24] (Table S5), was also performed; discontinuation rates are assumed to be identical for patients with and without AEs as data on AEs were not available.

## Results

### Base-case analysis

The results of the primary and secondary base-case analyses over a 5-year period are presented (Table 3). For the primary analysis, total costs per patient were lower with solifenacin 5 mg/day than tolterodine ER 4 mg/day (difference £23), and the solifenacin strategy was associated with a QALY gain (0.0066 QALYs). Solifenacin 5 mg/day was the dominant (i.e., more effective and less costly) treatment strategy versus tolterodine ER 4 mg/day.

**Table 3.** Cost-effectiveness analysis of solifenacin 5 mg/day compared with other oral antimuscarinic agents at 5 years (base-case scenario).

	Primary analysis		Secondary analysis						
	Solifenacin 5 mg/day	Tolterodine ER 4 mg/day	Fesoterodine 4 mg/day	Fesoterodine 8 mg/day	Oxybutynin ER 10 mg/day	Oxybutynin IR 10 mg/day	Solifenacin 10 mg/day	Tolterodine IR 4 mg/day	Trospium chloride 60 mg/day
Cost, £									
Drug acquisition	267	280	283	205	249	16	250	25	211
Other OAB drug(s)	359	355	354	376	363	381	380	365	367
GP visits	206	204	204	212	207	213	213	208	209
Specialist visits	446	443	443	459	450	463	462	451	453
Botulinum toxin	136	134	133	145	138	147	147	139	140
Incontinence pads	320	339	331	339	330	334	327	340	322
Total costs, £	1,733	1,756	1,748	1,736	1,737	1,554	1,778	1,528	1,702
Total QALYs	3.732	3.725	3.727	3.723	3.728	3.724	3.728	3.723	3.730
Incremental costs, £	‒	–23	‒15	‒3	‒5	179	‒46	205	31
Incremental QALYs	‒	0.0066	0.0043	0.0089	0.0033	0.0080	0.0041	0.0085	0.0020
ICER, £ per QALY gained	‒	Dominant	Dominant	Dominant	Dominant	22,393	Dominant	23,975	15,007

ER, extended release; GP general practitioner; ICER, incremental cost-effectiveness ratio; IR, immediate release; OAB, overactive bladder; QALY, quality-adjusted life year.

In the secondary analysis, solifenacin 5 mg/day was also dominant versus fesoterodine 4 and 8 mg/day, oxybutynin ER 10 mg/day, and solifenacin 10 mg/day. Solifenacin 5 mg/day was also cost-effective versus oxybutynin IR 10 mg/day (ICER, £22,393 per QALY), tolterodine IR 4 mg/day (ICER, £23,975 per QALY) and trospium chloride 60 mg/day (ICER, £15,007 per QALY).

### Sensitivity analyses

#### Deterministic sensitivity analyses

**Figure 1.**
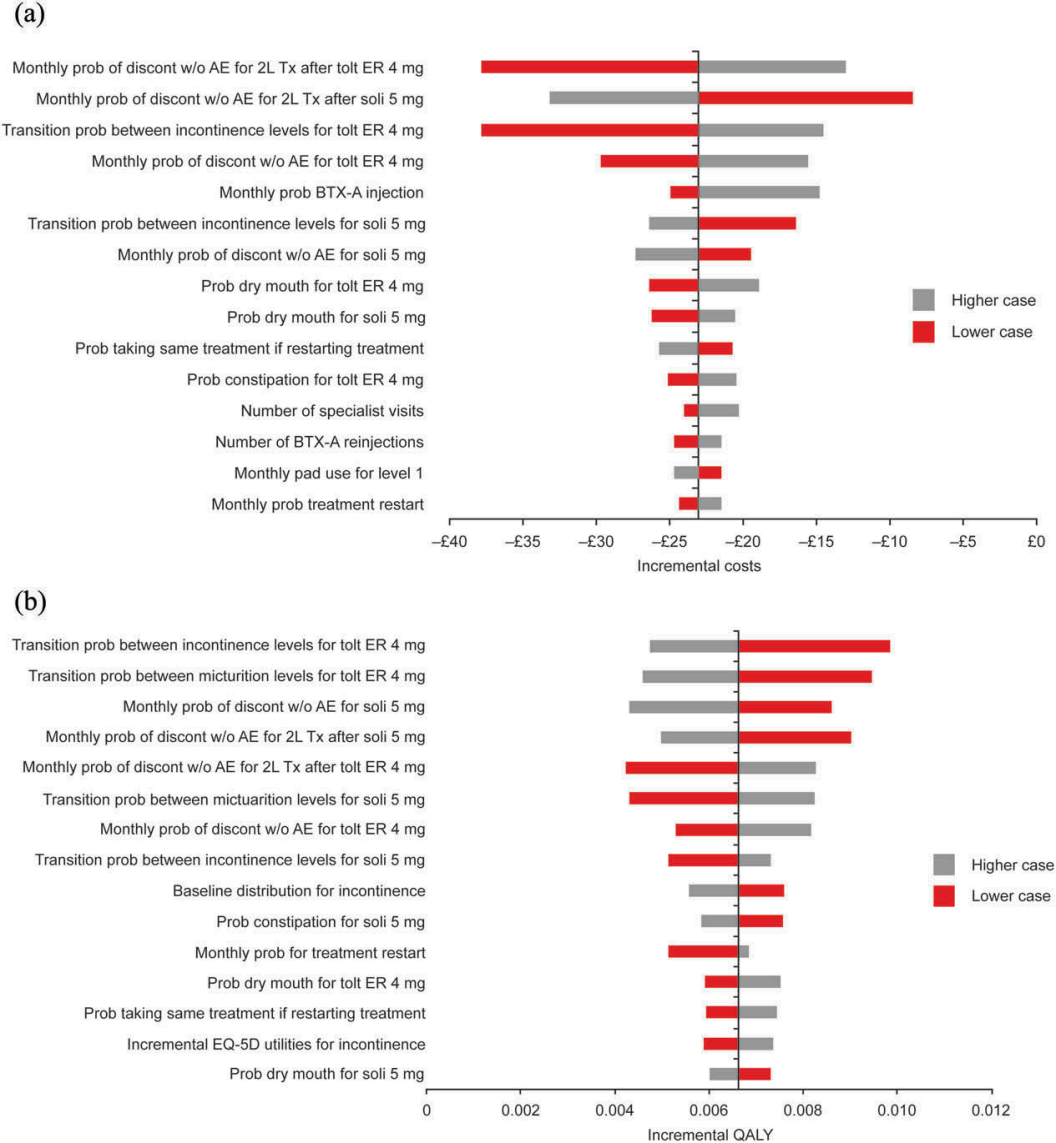
Deterministic sensitivity analysis: Tornado diagram showing the effects of varying key input parameters on (**A**) costs and (**B**) QALYs for solifenacin 5 mg/day versus tolterodine ER 4 mg/day. AE, adverse event; BTX-A, botulinum toxin type A; discon, discontinuation; ER, extended release; ICER, incremental cost-effectiveness ratio; QALY, quality-adjusted life year; prob, probability; soli, solifenacin; tolt, tolterodine; Tx, treatment; w/o, without; 2L, second-line.

Tornado diagrams for costs and QALYs for solifenacin 5 mg/day versus tolterodine ER 4 mg/day are shown in Figure 1. The model was generally most sensitive to the probability of treatment discontinuation, transition probabilities between symptom severity levels, the baseline distribution of patients across severity levels and the probability of dry mouth.

#### Probabilistic sensitivity analyses

**Figure 2.**
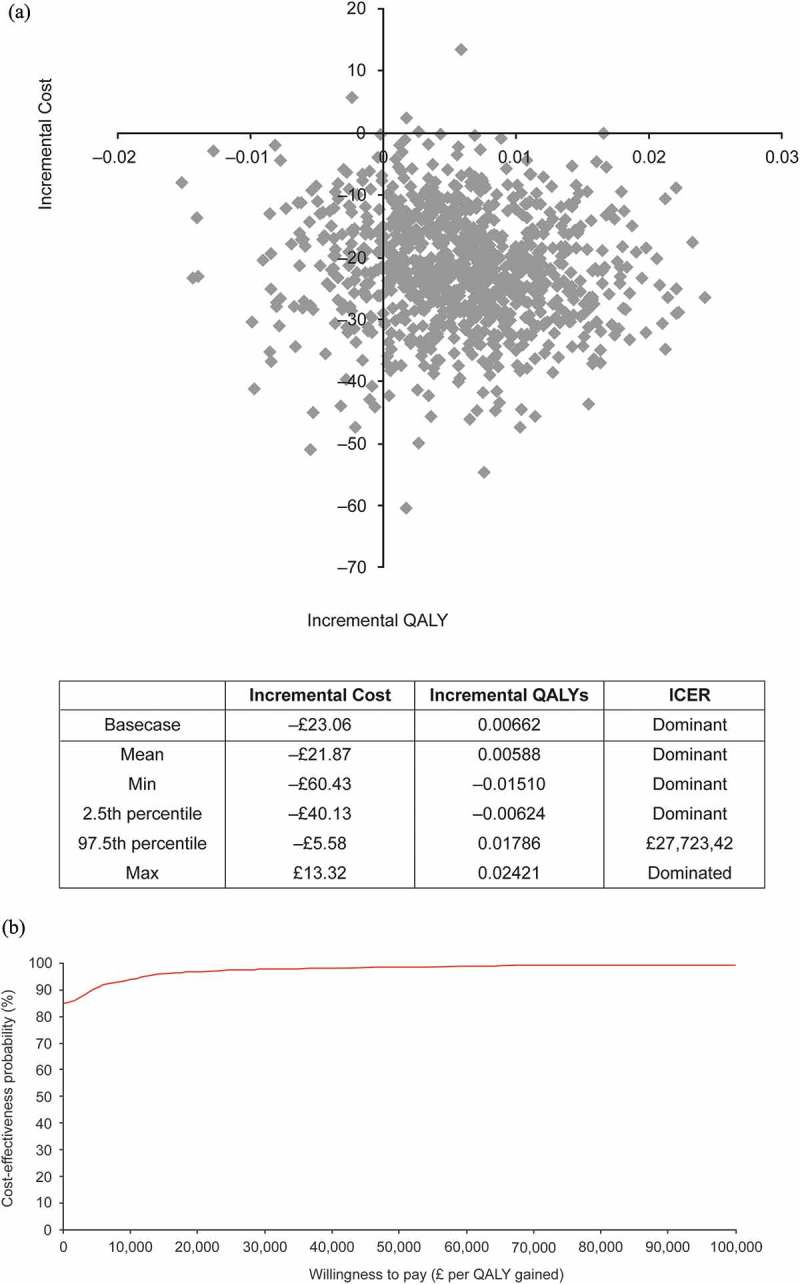
Probabilistic sensitivity analysis: (**A**) cost-effectiveness plane and (**B**) cost-effectiveness acceptability curve for solifenacin 5 mg/day versus tolterodine ER 4 mg/day. ER, extended-release; ICER, incremental cost-effectiveness ratio; QALY, quality-adjusted life year.

The model was robust to simultaneous variations of model inputs. The cost-effectiveness plane and cost-effectiveness acceptability curve for solifenacin 5 mg/day versus tolterodine ER 4 mg/day are shown in Figure 2. At a WTP threshold of £30,000/QALY, the probability that solifenacin 5 mg/day was cost-effective versus tolterodine ER 4 mg/day was 93.3%, and versus other comparators was 94.2% (fesoterodine 4 mg/day), 98.3% (fesoterodine 8 mg/day), 86.6% (oxybutynin ER 10 mg/day), 66.8% (oxybutynin IR 10 mg/day), 93.3% (solifenacin 10 mg/day), 58.1% (tolterodine IR 4 mg/day), and 56.6% (trospium chloride 60 mg/day).

#### Scenario analysis

When discontinuation rates from a UK observational study were used as model inputs [24], the results were generally similar to the base-case analysis except that solifenacin 5 mg/day was cost-effective, rather than dominant, versus oxybutynin ER (ICER, £156 per QALY), and solifenacin 5 mg/day was less costly and less effective (ICER, £39,731.60 per QALY) rather than dominant, versus solifenacin 10 mg/day (Table S5).

## Discussion

This economic model shows that, over a 5-year time horizon, solifenacin 5 mg/day is dominant (i.e., lower costs combined with a health benefit) versus tolterodine ER 4 mg/day, fesoterodine 4 and 8 mg/day, oxybutynin ER 10 mg/day and solifenacin 10 mg/day, and cost-effective versus oxybutynin IR 10 mg/day, tolterodine IR 4 mg/day and trospium chloride 60 mg/day for the treatment of adults with OAB from a UK payer perspective. The ICERs for all comparators were below the upper limit of the WTP range generally applied by NICE in their health technology appraisals (£20,000–£30,000 per QALY) [17]. The probabilistic sensitivity analysis showed that, at a threshold of £30,000 per QALY, the probability of solifenacin 5 mg/day being cost effective exceeded 80% compared with fesoterodine 4 and 8 mg/day, tolterodine ER 4 mg/day, oxybutynin ER 4 mg/day and solifenacin 10 mg/day.

In our model, the results of the comparison with tolterodine ER 4 mg/day was driven primarily by differences in efficacy leading to QALY gains with solifenacin 5 mg/day, whereas for other comparators, the QALY gains and cost offsets were driven mainly by differences in AEs and persistence on treatment. Hence, the assumption that AEs are a key contributor to treatment discontinuation had an important role in our model. This assumption is supported by the literature, which shows that the most common reasons for discontinuing antimuscarinics are that treatment did not work as expected and side effects [35]. Further, better persistence rates with solifenacin compared with other commonly prescribed antimuscarinics have been observed in routine clinical practice in the UK and are consistent with our model predictions [24,36]. Given the key role played by discontinuation rates in our model, we replaced the assumed/derived values used in the base-case analysis with rates reported in a large UK observational study [24]. The results were largely unchanged compared with the base-case analysis, except that solifenacin 5 mg/day was cost-effective versus oxybutynin ER and solifenacin 10 mg/day rather than dominant.

Three other cost-effectiveness analyses have compared solifenacin with oral antimuscarinics in adults with OAB from a UK healthcare provider perspective with 1-year time horizons; one used a decision-tree analysis [32] and the other two [37,38] were based on an earlier Markov model [39]. In the decision-tree analysis [32], solifenacin 5/10 mg/day was found to be dominant compared with tolterodine ER 4 mg/day and fesoterodine 4/8 mg/day. Similarly, a flexible dosing schedule of solifenacin 5/10 mg/day was also dominant compared with tolterodine ER 4 mg/day [38]. In the third analysis, solifenacin 5/10 mg had an ICER of £12,309 compared with oxybutynin IR 15 mg/day [37], although AEs were not considered in this model. These studies support our own and suggest that solifenacin 5 mg/day, whether given as a fixed- or flexible-dose schedule, is a dominant treatment strategy in cost-effectiveness terms compared with tolterodine ER 4 mg/day in the UK.

A strength of our analysis is that it compared solifenacin with antimuscarinics or formulations not considered in previous analyses. It is based on a new Markov state-transition model [14,15], which was then adapted for the present study. Mirabegron, another pertinent comparator, has been considered in other previously reported analyses based on the same model [14,15,40]. Unlike previous models described above, this model had a longer time horizon of 5 years, included treatment switching and treatment restart, as well as considering the effects of AEs on persistence and utilities. As OAB is known to be a chronic disorder with persistent symptoms [41], our model goes some way to simulating the expected long-term outcomes and costs arising from this condition. Another strength of our model is that the utilities were based on the EQ-5D, the instrument preferred by NICE for cost-utility analyses [42], and were estimated using data from a large trial of OAB patients. This cost-effectiveness analysis focuses on clinical practice in the UK and the results are potentially relevant in other countries because of consistency between OAB treatment guidelines, although we acknowledge that variations in healthcare practice may affect direct costs and the cost-effectiveness of specific interventions.

Limitations of our model included the use of indirect evidence from an NMA to estimate the relative efficacy and tolerability of solifenacin versus other antimuscarinics. Transition probabilities for comparators were determined by calibration, which led to uncertainty around these inputs. We also acknowledge that the model treatment pathway may have been over-simplified, e.g., solifenacin 10 mg/day was evaluated as an alternative to solifenacin 5 mg/day, but in reality is likely to be used as part of a flexible-dose schedule with solifenacin 5 mg/day. The model considered micturition and incontinence as symptoms of OAB, but excluded urgency and nocturia. The instruments used to measure urgency are subjective and have different severity thresholds, which would have introduced further uncertainty. Nocturia is multifactorial and not always related to OAB alone, so it was considered appropriate to omit both symptoms from the model.

Current NICE guidelines for urinary incontinence in women recommend oxybutynin IR, tolterodine IR and darifenacin as initial treatments followed by another drug with a low acquisition cost if the first treatment is either not effective or poorly tolerated [7]. In our analysis, solifenacin 5 mg/day was cost-effective compared with oxybutynin IR and tolterodine IR (ICER £22,000 and £24,000 per QALY gained, respectively). We were unable to include darifenacin in the present model because it was not possible to estimate its relative effectiveness for incontinence. Our model emphasizes that drug acquisition cost is only one of several costs accrued when treating patients, and can be offset by treatment benefits, such as symptomatic and HRQoL improvements.

## Conclusions

Solifenacin 5 mg/day appears to be a cost-effective strategy compared with other oral antimuscarinic agents commonly used for the treatment of adults with OAB from a UK NHS perspective. By providing a comparative assessment of the cost effectiveness of oral antimuscarinic agents commonly used in the UK, these findings will help to inform decisions made by both clinicians and healthcare policy makers regarding treatment options for OAB.

## References

[1] Abrams P, Cardozo L, Fall M, et al The standardisation of terminology in lower urinary tract function: report from the standardisation sub-committee of the International Continence Society. Urology. 2003;61:37–11. 12559262 10.1016/s0090-4295(02)02243-4

[2] Coyne KS, Sexton CC, Irwin DE, et al The impact of overactive bladder, incontinence and other lower urinary tract symptoms on quality of life, work productivity, sexuality and emotional well-being in men and women: results from the EPIC study. BJU Int. 2008;101:1388–1395. 18454794 10.1111/j.1464-410X.2008.07601.x

[3] Irwin DE, Milsom I, Hunskaar S, et al Population-based survey of urinary incontinence, overactive bladder, and other lower urinary tract symptoms in five countries: results of the EPIC study. Eur Urol. 2006;50:1306–1314. 17049716 10.1016/j.eururo.2006.09.019

[4] Irwin DE, Kopp ZS, Agatep B, et al Worldwide prevalence estimates of lower urinary tract symptoms, overactive bladder, urinary incontinence and bladder outlet obstruction. BJU Int. 2011;108:1132–1138. 21231991 10.1111/j.1464-410X.2010.09993.x

[5] Irwin DE, Mungapen L, Milsom I, et al The economic impact of overactive bladder syndrome in six Western countries. BJU Int. 2009;103:202–209. 19278532 10.1111/j.1464-410X.2008.08036.x

[6] National Institute for Health and Care Excellence CG97. The management of lower urinary tract symptoms in men: NICE guideline; 2010 May [cited 2017 Aug] Available from: https://www.nice.org.uk/guidance/cg97 31909931

[7] National Institute for Health and Care Excellence CG171. The management of urinary incontinence in women: NICE guideline; 2013 Sep [cited 2017 Aug] Available from: https://www.nice.org.uk/guidance/cg171

[8] Solifenacin (Vesicare®) Summary of Product Characteristics 2013 [cited 2017 Aug] Available from: https://www.medicines.org.uk/emc/medicine/14900

[9] Chapple CR, Martinez-Garcia R, Selvaggi L, et al A comparison of the efficacy and tolerability of solifenacin succinate and extended release tolterodine at treating overactive bladder syndrome: results of the STAR trial. Eur Urol. 2005;48:464–470. 15990220 10.1016/j.eururo.2005.05.015

[10] Chapple CR, Rechberger T, Al-Shukri S, et al Randomized, double-blind placebo- and tolterodine-controlled trial of the once-daily antimuscarinic agent solifenacin in patients with symptomatic overactive bladder. BJU Int. 2004;93:303–310. 14764127 10.1111/j.1464-410x.2004.04606.x

[11] Madhuvrata P, Cody JD, Ellis G, et al Which anticholinergic drug for overactive bladder symptoms in adults. Cochrane Database Syst Rev. 2012;18(1):CD005429. 10.1002/14651858.CD005429.pub2 PMC12989262 22258963

[12] Luo D, Liu L, Han P, et al Solifenacin for overactive bladder: a systematic review and meta-analysis. Int Urogynecol J. 2012;23:983–991. 22310924 10.1007/s00192-011-1641-7

[13] Nazir J, Kelleher C, Aballéa S, et al Comparative efficacy and tolerability of solifenacin 5 mg/day versus oral antimuscarinic agents in overactive bladder: a systematic literature review and network meta-analysis. Neurourol Urodyn. 2017;1–11. DOI:10.1002/nau.23413 29140559

[14] Aballéa S, Maman K, Thokagevistk K, et al Cost effectiveness of mirabegron compared with tolterodine extended release for the treatment of adults with overactive bladder in the United Kingdom. Clin Drug Investig. 2015;35:83–93. 10.1007/s40261-014-0240-z PMC4300413 25491433

[15] Nazir J, Maman K, Neine ME, et al Cost-effectiveness of mirabegron compared with antimuscarinic agents for the treatment of adults with overactive bladder in the United Kingdom. Value Health. 2015;18:783–790. 26409605 10.1016/j.jval.2015.05.011

[16] Sonnenberg FA, Beck JR. Markov models in medical decision making: a practical guide. Med Decis Making. 1993;13:322–338. 8246705 10.1177/0272989X9301300409

[17] National Institute for Health and Care Excellence PMG9. Guide to the methods of technology appraisal 2013: NICE article; 2013 Apr [cited 2017 Aug] Available from: http://www.nice.org.uk/article/pmg9/chapter/foreword 27905712

[18] Jansen JP, Crawford B, Bergman G, et al Bayesian meta-analysis of multiple treatment comparisons: an introduction to mixed treatment comparisons. Value Health. 2008;11:956–964. 18489499 10.1111/j.1524-4733.2008.00347.x

[19] Van Kerrebroeck PE, Heesakkers J, Berriman S, et al Long-term safety, tolerability and efficacy of fesoterodine treatment in subjects with overactive bladder symptoms. Int J Clin Pract. 2010;64:584–593. 20201992 10.1111/j.1742-1241.2010.02361.x

[20] Chapple CR, Kaplan SA, Mitcheson D, et al Randomized double-blind, active-controlled phase 3 study to assess 12-month safety and efficacy of mirabegron, a β(3)-adrenoceptor agonist, in overactive bladder. Eur Urol. 2013;63:296–305. 23195283 10.1016/j.eururo.2012.10.048

[21] Vanni T, Karnon J, Madan J, et al Calibrating models in economic evaluation: a seven-step approach. Pharmacoeconomics. 2011;29:35–49. 21142277 10.2165/11584600-000000000-00000

[22] Astellas Pharma Europe Ltd Solifenacin. Study 905-CL-015: number and percentage of patients with at least one TEAE, classified by System Organ Class and Preferred Term. Data on file. 2016.

[23] Castro-Diaz D, Miranda P, Sanchez-Ballester F, et al Assessment of reasons for overactive bladder treatment change [Article in Spanish]. Actas Urol Esp. 2011;35:73–79. 21296454 10.1016/j.acuro.2010.11.011

[24] Chapple CR, Nazir J, Hakimi Z, et al Persistence and adherence with mirabegron versus antimuscarinic agents in patients with overactive bladder: a retrospective observational study in UK clinical practice. Eur Urol. 2017;72:389–399. 28196724 10.1016/j.eururo.2017.01.037

[25] Odeyemi IA, Dakin HA, O’Donnell RA, et al Epidemiology, prescribing patterns and resource use associated with overactive bladder in UK primary care. Int J Clin Pract. 2006;60:949–958. 16893437 10.1111/j.1742-1241.2006.01057.x

[26] Khullar V, Amarenco G, Angulo JC, et al Efficacy and tolerability of mirabegron, a β(3)-adrenoceptor agonist, in patients with overactive bladder: results from a randomised European-Australian phase 3 trial. Eur Urol. 2013;63:283–295. 23182126 10.1016/j.eururo.2012.10.016

[27] Dolan P Modeling valuations for EuroQol health states. Med Care. 1997;35:1095–1108. 9366889 10.1097/00005650-199711000-00002

[28] Joint Formulary Committee British National Formulary. London: BMJ Group and Pharmaceutical Press; 2016 9.

[29] Curtis L, Burns A Personal Social Services Research Unit (PSSRU). Unit Costs of Health and Social Care; 2015 [cited 2017 Aug] Available from: http://www.pssru.ac.uk/project-pages/unit-costs/2015/#sections

[30] Department of Health NHS reference costs 2014–15; [cited 2017 Aug] Available from: https://www.gov.uk/government/publications/nhs-reference-costs-2014-to-2015

[31] Age UK incontinence Lille Healthcare Classic Form Regular 1300ml pack of 28; [cited 2017 Aug] Available from: http://www.ageukincontinence.co.uk

[32] Cardozo L, Thorpe A, Warner J, et al The cost-effectiveness of solifenacin vs fesoterodine, oxybutynin immediate-release, propiverine, tolterodine extended-release and tolterodine immediate-release in the treatment of patients with overactive bladder in the UK National Health Service. BJU Int. 2010;106:506–514. 20132203 10.1111/j.1464-410X.2009.09160.x

[33] Astellas Pharma Europe Ltd Solifenacin. Mean number of pads/day per incontinence severity level estimated from Study 905-CL-015 data. Data on file. 2016.

[34] Dakin H, Devlin N, Feng Y, et al The influence of cost-effectiveness and other factors on NICE decisions. Health Econ. 2014;23 DOI:10.1002/hec.3086 25251336

[35] Sexton CC, Notte SM, Maroulis C, et al Persistence and adherence in the treatment of overactive bladder syndrome with anticholinergic therapy: a systematic review of the literature. Int J Clin Pract. 2011;65:567–585. 21489081 10.1111/j.1742-1241.2010.02626.x

[36] Wagg A, Compion G, Fahey A, et al Persistence with prescribed antimuscarinic therapy for overactive bladder: a UK experience. BJU Int. 2012;110:1767–1774. 22409769 10.1111/j.1464-410X.2012.11023.x

[37] Hart WM, Abrams P, Munro V, et al Cost-effectiveness analysis of solifenacin versus oxybutynin immediate-release in the treatment of patients with overactive bladder in the UK. J Med Econ. 2013;16:1246–1254. 23885660 10.3111/13696998.2013.829079

[38] Speakman M, Khullar V, Mundy A, et al A cost-utility analysis of once daily solifenacin compared to tolterodine in the treatment of overactive bladder syndrome. Curr Med Res Opin. 2008;24:2173–2179. 18565239 10.1185/03007990802234829

[39] Kobelt G, Jönsson L, Mattiasson A Cost-effectiveness of new treatments for overactive bladder: the example of tolterodine, a new muscarinic agent: a Markov model. Neurourol Urodynam. 1998;17:599–611. 10.1002/(sici)1520-6777(1998)17:6<599::aid-nau4>3.0.co;2-j 9829424

[40] Herschorn S, Nazir J, Ramos B, et al Cost-effectiveness of mirabegron compared to tolterodine ER 4 mg for overactive bladder in Canada. Can Urol Assoc J. 2017;11:123–130. 28515812 10.5489/cuaj.4114 PMC5434500

[41] Garnett S, Abrams P The natural history of the overactive bladder and detrusor overactivity. A review of the evidence regarding the long-term outcome of the overactive bladder. J Urol. 2003 3;169:843–848. 12576796 10.1097/01.ju.0000050305.05345.40

[42] National Institute for Health and Care Excellence The guidelines manual Process and methods [PMG6]; 2012 Nov [cited 2017 Aug] Available from: https://www.nice.org.uk/process/pmg6/chapter/assessing-cost-effectiveness

