# Supplementary material for: Cost-effectiveness of solifenacin compared with oral antimuscarinic agents for the treatment of patients with overactive bladder in the UK
Source: J Mark Access Health Policy. 2018 Mar 20;6(1):1438721. doi: 10.1080/20016689.2018.1438721 (PMC5907635; doi:10.1080/20016689.2018.1438721)

# Supplemental Information

Table S1. Network meta-analyses for solifenacin 5 mg/day versus antimuscarinic agents: efficacy and safety outcomes 2

Table S2. Symptom severity levels: definitions and distribution of patients at baseline 3

Table S3. Transition probabilities for solifenacin 5 mg/day between symptom severity levels 4

Table S4. Utility values derived from EQ-5D index scores for each health state 5

Table S5. Scenario analyses: cost-effectiveness of solifenacin 5 mg/day compared with other oral antimuscarinic agents at 5 years using persistence rates from a UK observational study 6

Figure S1. Model structure: treatment pathways 7

**TABLE S1.** **Network meta-analysis for solifenacin 5 mg/day versus antimuscarinic agents: efficacy and safety outcomes^1^**

| **Treatment** | **Treatment effect (mean difference)^a^** | | **Adverse events (odds ratio)^b^** | |
| --- | --- | --- | --- | --- |
|  | **Micturition** | **Incontinence** | **Dry mouth** | **Constipation** |
| Tolterodine 4 mg/day^c^ | 0.157 | 0.297 | ‒ | ‒ |
| Tolterodine ER^c^ | ‒ | ‒ | 1.046 | 0.585 |
| Tolterodine IR^c^ | ‒ | ‒ | 1.720 | 0.491 |
| Fesoterodine 4 mg/day | 0.223 | 0.239 | 1.062 | 0.501 |
| Fesoterodine 8 mg/day | ‒0.043 | 0.360 | 2.44 | 0.961 |
| Oxybutynin 10 mg/day^c^ | ‒0.018 | 0.159 | ‒ | ‒ |
| Oxybutynin ER^c^ | ‒ | ‒ | 1.594 | 0.498 |
| Oxybutynin IR^c^ | ‒ | ‒ | 3.539 | 0.495 |
| Solifenacin 10 mg/day | ‒0.261 | ‒0.058 | 2.274 | 1.792 |
| Trospium chloride 60 mg/day | 0.053 | ‒0.053^d^ | 0.870 | 2.289 |

ER, extended release; IR, immediate release.

^a^Mean change from baseline at 3 months versus solifenacin 5 mg/day; a positive value favors solifenacin. ^b^Compared with solifenacin 5 mg/day; odds ratio >1 favors solifenacin 5 mg/day and <1 favors the comparator. ^c^ER and IR formulations were assumed to have similar efficacy and were not separated for efficacy outcomes, but were presented separately for tolerability outcomes. ^d^Improvement in urge incontinence was used instead of incontinence improvement for patients treated with trospium chloride 60 mg since the mean change in frequency of incontinence episodes was not reported in studies.

**Reference**

1. Nazir J, Kelleher C, Aballéa S, et al. Comparative efficacy and tolerability of solifenacin 5 mg/day versus oral antimuscarinic agents in overactive bladder: a systematic literature review and network meta-analysis. Manuscript submitted to Neurourol Urodyn.

**TABLE S2. Symptom severity levels: definitions and distribution of patients at baseline**

| **Symptom severity** | **Micturition** | | **Incontinence** | |
| --- | --- | --- | --- | --- |
|  | **Micturitions/day, mean (n)** | **Proportion of patients, %** | **Incontinence episodes/day, mean (n)** | **Proportion of patients, %** |
| Level 1 | ≤8 | 6.20 | 0 | 41.05 |
| Level 2 | >8–≤10 | 29.62 | 1 | 20.91 |
| Level 3 | >10–≤12 | 27.11 | 2 | 12.58 |
| Level 4 | >12–≤14 | 15.39 | 3 | 10.16 |
| Level 5 | >14 | 21.68 | >3 | 15.30 |

Note: The initial proportions of patients at different symptom severity levels were obtained from 905‑CL‑015^1^ based on pooled data from the treatment arms at baseline.

**Reference**

1. Chapple CR, Rechberger T, Al-Shukri S, et al. Randomized, double-blind placebo- and tolterodine-controlled trial of the once-daily antimuscarinic agent solifenacin in patients with symptomatic overactive bladder. BJU Int 2004;93:303-10.

**TABLE S3.** **Transition probabilities for solifenacin 5 mg/day between symptom severity levels**

|  | | **Level 1** | **Level 2** | **Level 3** | **Level 4** | **Level 5** |
| --- | --- | --- | --- | --- | --- | --- |
| **Micturition** | | **Severity level at 1 month** | | | | |
| Severity level at baseline | 1 | 0.777 | 0.202 | 0.015 | 0.006 | 0.000 |
|  | 2 | 0.407 | 0.470 | 0.104 | 0.017 | 0.002 |
|  | 3 | 0.180 | 0.425 | 0.298 | 0.086 | 0.011 |
|  | 4 | 0.067 | 0.311 | 0.304 | 0.225 | 0.093 |
|  | 5 | 0.047 | 0.064 | 0.165 | 0.234 | 0.490 |
|  | | **Severity level at 2 months** | | | | |
| Severity level at 1 month | 1 | 0.688 | 0.267 | 0.032 | 0.013 | 0.000 |
|  | 2 | 0.290 | 0.499 | 0.174 | 0.031 | 0.007 |
|  | 3 | 0.101 | 0.356 | 0.393 | 0.125 | 0.025 |
|  | 4 | 0.030 | 0.210 | 0.324 | 0.265 | 0.171 |
|  | 5 | 0.015 | 0.030 | 0.124 | 0.194 | 0.637 |
|  | | **Severity level at 3 months** | | | | |
| Severity level at 2 months | 1 | 0.757 | 0.207 | 0.026 | 0.010 | 0.000 |
|  | 2 | 0.364 | 0.441 | 0.163 | 0.027 | 0.006 |
|  | 3 | 0.135 | 0.334 | 0.390 | 0.115 | 0.025 |
|  | 4 | 0.042 | 0.203 | 0.330 | 0.250 | 0.176 |
|  | 5 | 0.020 | 0.029 | 0.124 | 0.181 | 0.645 |
| **Incontinence** | | **Severity level at 1 month** | | | | |
| Severity level at baseline | 1 | 0.932 | 0.053 | 0.011 | 0.002 | 0.002 |
|  | 2 | 0.619 | 0.280 | 0.071 | 0.017 | 0.013 |
|  | 3 | 0.365 | 0.299 | 0.222 | 0.080 | 0.034 |
|  | 4 | 0.250 | 0.203 | 0.271 | 0.196 | 0.081 |
|  | 5 | 0.189 | 0.107 | 0.151 | 0.149 | 0.404 |

|  | | **Severity level at 2 months** | | | | |
| --- | --- | --- | --- | --- | --- | --- |
| Severity level at 1 month | 1 | 0.897 | 0.072 | 0.021 | 0.004 | 0.005 |
|  | 2 | 0.502 | 0.319 | 0.120 | 0.030 | 0.030 |
|  | 3 | 0.241 | 0.277 | 0.306 | 0.111 | 0.065 |
|  | 4 | 0.143 | 0.164 | 0.324 | 0.237 | 0.132 |
|  | 5 | 0.089 | 0.071 | 0.148 | 0.149 | 0.543 |
|  | | **Severity level at 3 months** | | | | |
| Severity level at 2 months | 1 | 0.897 | 0.072 | 0.021 | 0.004 | 0.005 |
|  | 2 | 0.502 | 0.319 | 0.120 | 0.030 | 0.030 |
|  | 3 | 0.241 | 0.277 | 0.306 | 0.111 | 0.065 |
|  | 4 | 0.143 | 0.164 | 0.324 | 0.237 | 0.132 |
|  | 5 | 0.089 | 0.071 | 0.148 | 0.149 | 0.543 |

**TABLE S4.** **Utility values derived from EQ-5D index scores for each health state**

| **Incontinence severity level** | **Micturition severity level** | | | | |
| --- | --- | --- | --- | --- | --- |
|  | **1** | **2** | **3** | **4** | **5** |
| 1 | 0.85 | 0.83 | 0.81 | 0.80 | 0.79 |
| 2 | 0.83 | 0.81 | 0.79 | 0.78 | 0.77 |
| 3 | 0.82 | 0.80 | 0.78 | 0.77 | 0.76 |
| 4 | 0.80 | 0.78 | 0.76 | 0.75 | 0.74 |
| 5 | 0.79 | 0.77 | 0.75 | 0.74 | 0.73 |

EQ-5D, European Quality of Life 5 Dimensions.

Note: 0 = worst imaginable health and 1 = perfect health.

**TABLE S5.** **Scenario analysis: cost-effectiveness of solifenacin 5 mg/day compared with other oral antimuscarinic agents at 5 years using persistence rates from a UK observational study^1^**

| **Comparator** | **12-month persistence rate, %** | **Incremental costs, £** | **Incremental QALYs** | **ICER, £ per QALY gained** |
| --- | --- | --- | --- | --- |
| Tolterodine ER 4mg | 20.4 | ‒15.89 | 0.01029 | Dominant |
| Fesoterodine 4 mg | 24.0 | ‒13.08 | 0.00662 | Dominant |
| Fesoterodine 8 mg | 24.0 | ‒19.33 | 0.00782 | Dominant |
| Oxybutynin IR 10 mg | 12.4 | 207.26 | 0.00996 | 20,803.27 |
| Oxybutynin ER 10 mg | 17.2 | 1.22 | 0.00784 | 156.06 |
| Solifenacin 10 mg | 24.8 | ‒91.78 | ‒0.00231 | 39,731.60 |
| Tolterodine IR 4mg | 20.6 | 234.48 | 0.01068 | 21,951.65 |
| Trospium chloride 60mg | 19.1 | 36.19 | 0.00312 | 11,582.34 |

ER, extended release; ICER, incremental cost-effectiveness ratio; IR, immediate release; QALY, quality-adjusted life year.

**Reference**

1. Chapple CR, Nazir J, Hakimi Z, et al. Persistence and adherence with mirabegron versus antimuscarinic agents in patients with overactive bladder: a retrospective observational study in UK clinical practice. Eur Urol 2017;Epub ahead of print. DOI: 10.1016/j.eururo.2017.01.037.

**Figure. S1.** Model structure: treatment pathways.

OAB, overactive bladder.


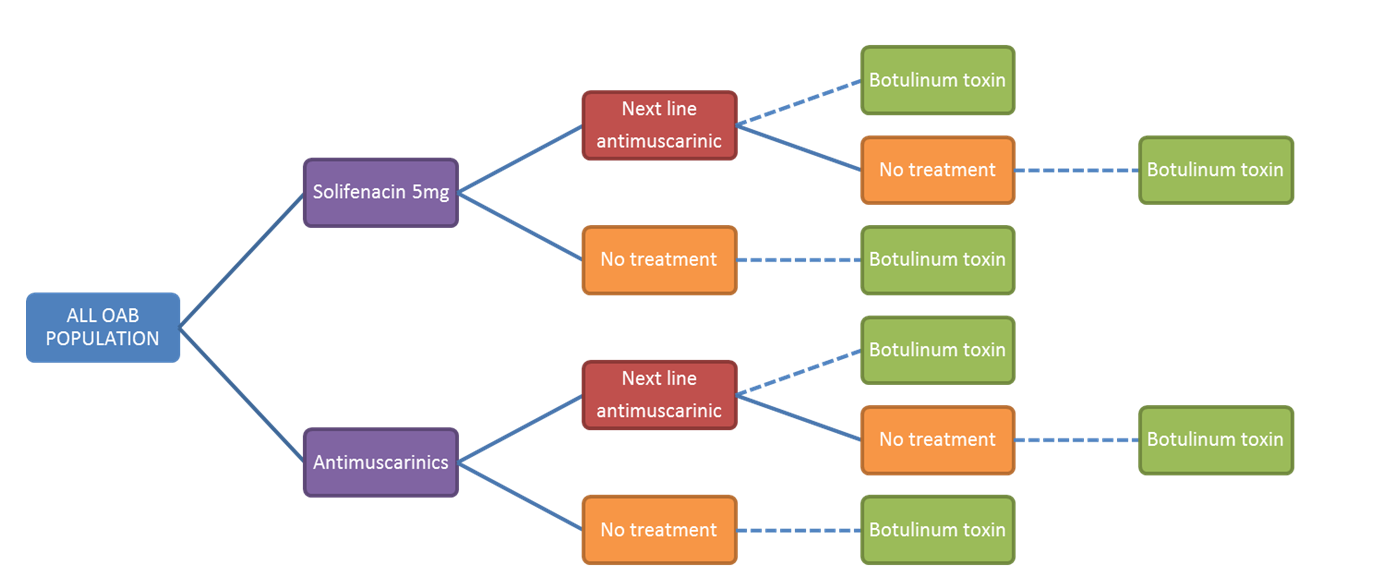

Supplement: Solifenacin_CEA_Supplemental_information_-_JMAHP.docx [file ZJMA_A_1438721_SM8291.docx]
